# The mucolipidosis III-causing mutation in *GNPTAB,* c.1760G>C, disrupts the development of somites in rats

**DOI:** 10.1016/j.gendis.2023.101172

**Published:** 2023-11-17

**Authors:** Tianying Nong, Jiangui Li, Xia Li, Yiqiang Li, Zhaohui Li, Weizhe Shi, Qiuchan Zhou, Hongwen Xu, Mingwei Zhu, Ya-Ping Tang

**Affiliations:** a Guangzhou Institute of Pediatrics, Guangzhou Women and Children's Medical Center, Guangzhou Medical University, Guangdong Provincial Clinical Research Center for Child Health, Guangzhou, Guangdong 510623, China; b Department of Pediatric Orthopedics, Guangzhou Women and Children's Medical Center, Guangdong Provincial Clinical Research Center for Child Health, Guangzhou, Guangdong 510623, China

Mucolipidosis (ML) II (OMIM 252500) and III α/β (OMIM 252600) are a group of rare lysosomal storage disorders caused by mis-sorting of lysosomal hydrolases and the subsequent accumulation of nondegraded macromolecules. These disorders manifest as multiple-systemic abnormalities throughout the body, mainly including severe dysplasia, short stature, scoliosis, joint stiffness, joint contractures, claw-hand deformities, craniofacial deformities, retinal degeneration, mental retardation, cognitive impairment, and internal organ dysfunction. ML II has an early onset, and patients usually die in early childhood. For ML III α/β, symptoms are less severe due to residual lysosomal enzymes, with a late onset and slow progression; these patients usually die in adulthood.^1^ Both ML II and ML III α/β are inherited in an autosomal recessive manner and caused by mutations in *GNPTAB*.

The mannose 6-phosphate-targeting signal is required for more than 50 hydrolases to be efficiently transported to lysosomes to catalyze the degradation of a variety of endogenous and exogenous macromolecules. By catalyzing the transfer of GlcNAc-1-phosphate from UDP-GlcNAc to mannose residues, the α_2_β_2_γ_2_ hexameric complex N-acetylglucosamine-1-phosphotransferase (GlcNAc-phosphotransferase, EC 2.7.8.17) is responsible for the initial step of mannose 6-phosphate modification. Mature α- and β-subunits originate from a common α/β-subunit precursor protein encoded by *GNPTAB*; the γ subunit is encoded by *GNPTG.* Functional loss of GlcNAc-phosphotransferase due to mutations in *GNPTAB* or *GNPTG* can lead to a lack of mannose 6-phosphate modification on lysosomal hydrolases, resulting in a decrease in lysosomal hydrolases targeted toward lysosomes and subsequent accumulation of nondegraded substances and ML II, ML III α/β, or ML III γ.^2^ According to the Human Gene Mutation Database, more than 330 different *GNPTAB* mutations to date have been associated with ML (https://www.hgmd.cf.ac.uk). Currently, there is no specific radical treatment for ML II and ML III; thus, accurate prenatal diagnosis through genetic screening is of great significance.

A one-and-a-half-year-old girl was brought to the pediatric orthopedics clinic due to kyphosis and inefficient knee extension. The patient's parents and sister were healthy with no related clinical phenotype (Fig. 1A). The patient had an abnormal facial appearance, hard and rough skin, kyphosis of the spine, stiffness of the limb joints, dysplasia of the hip joint, and flexion deformity of both knees (Fig. 1B). There was no obvious abnormality in intelligence or growth. X-ray and spinal computed tomography analyses were performed. The results showed the vertebral bodies to be flat with “beak” protrusion; the thoracolumbar junction was kyphotic; the T12 vertebral body was moved forward; and the intervertebral space was widened slightly. The patient also developed dysplasia of the hip joint, as indicated by the expanded iliac wing, pointed base, flattened acetabulum, deformed and flattened femoral head, and coxa valgus. In addition, the femoral shafts of both lower limbs were slightly thickened, and the bilateral calcaneal was deformed (Fig. 1C). After one year, the patient was treated with plaster vest fixation due to aggravation of kyphosis. The kyphotic Cobb angle was corrected from 31.6° to 14° (Fig. 1D).

**Figure 1:**
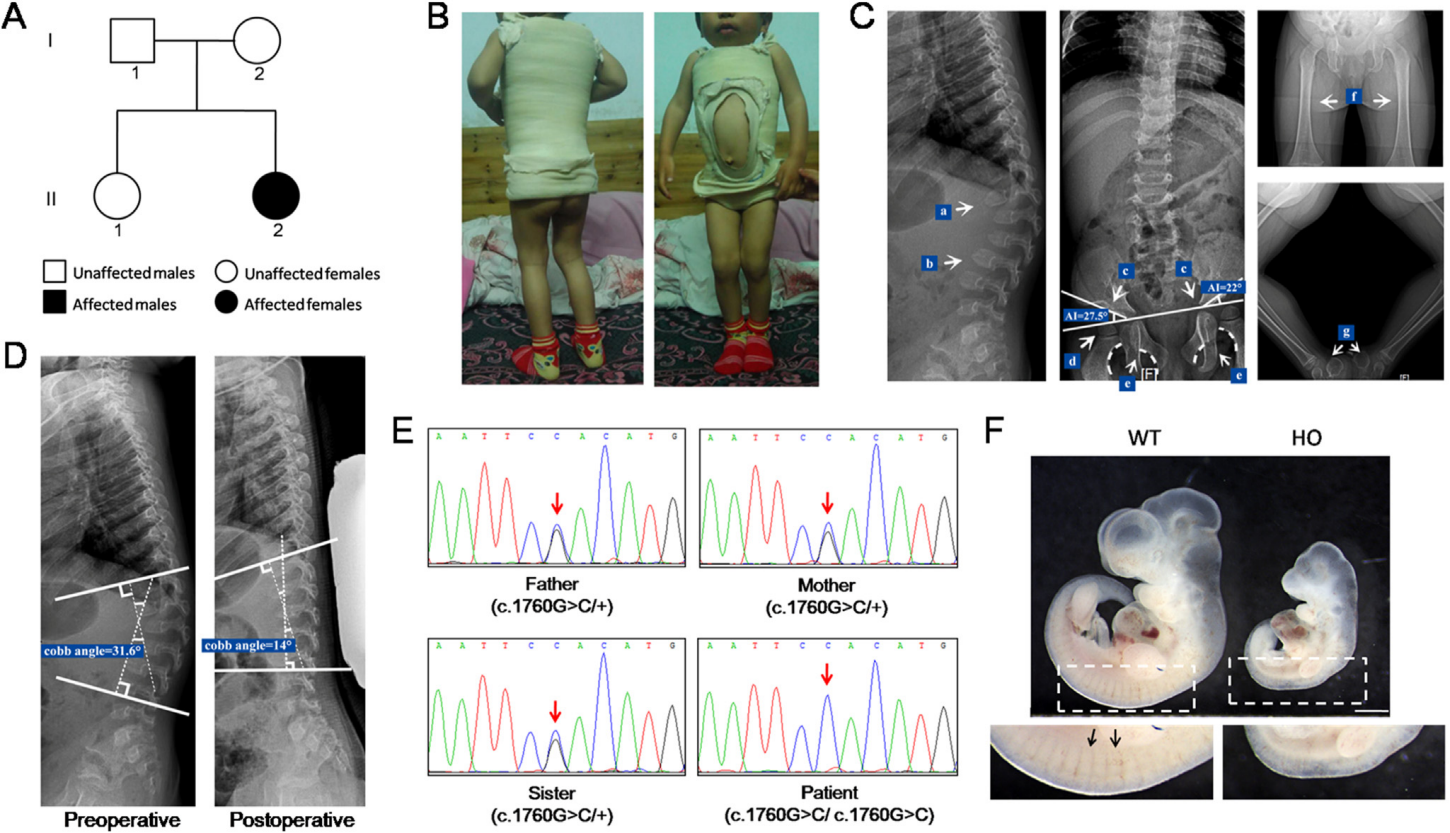
The *GNPTAB* c.1760G>C mutation identified in a ML III patient disrupts the development of somites in rats. **(A)** The pedigree of the patient (II:2). **(B)** The patient shows a rough face, kyphosis, and bilateral knee joint flexion deformity. **(C)** X-radiograph of the patient. Deformities are indicated by arrows. a) Kyphosis at the thoracolumbar junction. b) Flattened thoracic and lumbar vertebrae bodies with beak-like protrusions. c) Hip dysplasia with increased center-edge angle (right acetabular index = 27.5°, left acetabular index = 22°, normal acetabular index < 20°). d) Deformed and flattened femoral head. e) Disrupted Shenton's line. f) Slightly thickened femoral diaphysis in both lower limbs. g) Bilateral calcaneal malformation. **(D)** Treatment of kyphosis of the spine with plaster vest fixation. The kyphosis was significantly improved indicated by the corrected Cobb angle 6 months after surgery. **(E)** Sanger sequencing results from blood gDNA of family members. The patient (II:2) carries the homozygous mutation of *GNPTAB* c.1760G>C. The patient's parents and her sister all carry a heterozygous mutation. The red arrow points to the mutation site. **(F)** The size of the HO mutant embryo (embryonic day 10) is significantly smaller. The somites does not develop normally in the mutant embryos. WT, wild-type; HO, homozygote (*Gnptab*^*R587P/R587P*^). The arrows point to the somites in the WT embryo.

At the initial diagnosis, mucopolysaccharide diseases were considered due to multiple systemic abnormalities. Thus, levels of mucopolysaccharide disease-related lysosomal enzyme activity in plasma and mucopolysaccharide in urine were tested. All of the measurements were within the normal range, which led us to exclude mucopolysaccharide diseases (Table S1, 2). We then carried out whole-exome sequencing for the patient and her family members to aid in diagnosis. A homozygous mutation in exon 13 of *GNPTAB*, c.1760G>C (NM_024312.5), was detected in the patient, which was predicted to be deleterious by all prediction tools employed, including SIFT, FATHMM, Polyphen2, and MutationTaster (Table S3). Her parents and sister were heterozygous. This finding was verified by Sanger sequencing (Fig. 1E). *GNPTAB*, c.1760G>C is predicted to result in the substitution of an evolutionarily conserved amino acid residue 587 arginine by proline (p.R587P) in the α-subunit of GlcNAc-phosphotransferase (Fig. S1, 2). Arginine 587 is located in the γ-subunit binding domain (amino acids 536–698).^2^ We speculated that this mutation blocks the binding of the γ-subunit to the α/β-subunit, which reduces the activity of GlcNAc-phosphotransferase.^3^ As the patient had relatively mild clinical symptoms, we ultimately diagnosed her with ML III α/β.

*GNPTAB*, p.R587P was previously reported to be included in compound heterozygous mutations (p.R587P/p.D760EfsX12) detected in an ML III patient.^4^ According to the criteria of the American College of Medical Genetic and Genomics, this mutation is classified as “likely pathogenic” with 3 moderate (PM1, PM2, and PM3) and 3 supporting (PP3, PP4, and PP5) pathogenic evidence. To verify the pathogenicity of the p.R587P mutation, we generated a knock-in rat model that carried the corresponding mutation using a CRISPR-Cas9 gene editing system (Fig. S3 and Table S4). The heterozygous *Gnptab*-R587P knock-in rats were viable and bred normally. The heterozygotes were intercrossed to produce *Gnptab*^*R587P/R587P*^ homozygous offspring. Unfortunately, no homozygous offspring were obtained. As the patient carrying the *GNPTAB*^*R587P/R587P*^ homozygous mutation survived, we considered the effect of potential off-targets in the rat model. However, we could still not obtain homozygous offspring even when the heterozygous knock-in rats were crossed with wild-type for more than ten generations to clear the background. We found that all *Gnptab*^*R587P/R587P*^ rat embryos die at approximately embryonic day 10, with obvious abnormalities. The size of the mutant embryo was significantly smaller. The somites were not developed in mutant embryos, indicating an important role of GNPTAB in the development of somites, which might be the cause of their death (Fig. 1F). Although the phenotypes of ML III, such as skeletal dysplasia, cannot be recapitulated in the *Gnptab*^*R587P/R587P*^ rat model due to its early embryonic death, the results strongly indicate the pathogenicity of p.R587P mutation. It has been reported that mice harboring the p.G1028RfsX16 or p.Y867X homozygous mutation of *Gnptab* survive to adulthood and have ML-like phenotypes.^5^ We suggest that the lethality of *Gnptab*^*R587P/R587P*^ in rats may reflect the difference in GNPTAB function in different species or a deleterious gain-of-function effect of p.R587P.

In the present study, we first reported an ML III α/β case caused by the homozygous mutation c. 1760G>C (p.R587P) in *GNPTAB*. The mutation was identified through whole-exome sequencing in the patient and helped us make the ultimate diagnosis. The patient developed dysplasia of multiple systems, especially skeletal deformity primarily indicated by severe kyphosis of the spine and dysplasia of the hip joint. Treatment in our hospital with plaster vest fixation corrected kyphosis. Moreover, we generated a knock-in rat model that carried the p.R587P mutation. The developmental dysplasia and prenatal death of *Gnptab*^*R587P/R587P*^ rat embryos confirmed the pathogenicity of the p.R587P mutation. Our findings expand the genetic etiology spectrum of ML, which is of great significance for clinical genetic counseling and prenatal diagnosis of ML.

## Ethics declaration

The study was approved by the Human Ethics Committee of the Guangzhou Women and Children's Medical Center. Written informed consent was obtained from each participant or legal custodian.

## Author contributions

Y.-P.T., M.Z., and H.X. conceptualized and designed the study; J.L., Y.L., and W.S. obtained the clinical data; T.N., X.L., Z.L., and Q.Z. performed the experiments and analyzed the data; all authors interpreted and discussed the results. M.Z., T.N., and J.G.L. wrote the manuscript. All authors read and approved the final manuscript.

## Conflict of interests

The authors declare no conflict of interests.

## Funding

This work was supported by the 10.13039/501100001809National Natural Science Foundation of China (No. 81972038 to M.Z.) and the 10.13039/501100003453Natural Science Foundation of Guangdong Province, China (No. 2023A1515010281 to M.Z.), and partially by Guangzhou Municipal Science and Technology Project, Guangdong, China (No. 202201010838 to M.Z.).
