# Supplementary material for: The mucolipidosis III-causing mutation in GNPTAB, c.1760G>C, disrupts the development of somites in rats
Source: Genes Dis. 2023 Nov 17;11(6):101172. doi: 10.1016/j.gendis.2023.101172 (PMC11327505; doi:10.1016/j.gendis.2023.101172)
Supplement: Multimedia component 1 [file mmc1.docx]

**Materials and Methods**

**Subjects**

The patient, a one and a half year old girl, was referred to the pediatric orthopedics clinic of Guangzhou Women and Children’s Medical Center due to spine kyphosis and inefficient knee extension of both lower limbs. Physical and radiographic examinations were performed. Her family members, including her parents and her sister, were asymptomatic.

**Genomic DNA preparation and whole-exome sequencing**

Genomic DNA (gDNA) was extracted from the peripheral blood of the patient with a Blood DNA Kit (Omega, USA) according to the manufacturer’s instructions. Whole-exome sequencing (WES) was performed at Beijing Genomics Institute (Shenzhen, China). The data analysis was performed as previously described.^1^ Simply, the Genome Analysis Toolkit (GATK v3.3.0) was employed to detect single nucleotide variants and indels (SNV/INDEL). ANNOVAR software was used to annotate these variants. To identify the pathogenic variant, all variants were further filtered following a pipeline: 1) exclusion of variants with a frequency greater than 1% in any of the four databases, including 1000g_all, esp6500siv2_all, gnomAd_ALL and gnomAD_EAS; 2) exclusion of variants that were not in the coding (exonic) region or splicing region (splicing site±10 bp); 3) exclusion of synonymous SNPs that were not predicted by dbscSNV to affect splicing; and 4) retention of variants that were predicted by at least two of four prediction tools (SIFT, FATHMM, Polyphen2, and MutationTaster) to be deleterious and variants that were predicted to affect splicing.

**Variant verification**

The mutation in *GNPTAB* was further confirmed by Sanger sequencing. The target sequence was amplified by PCR with the following primers: 5’-CTGCACAAGGACGACATGC-3’ (forward) and 5’- TGAGACTCAACTGGGCGTC-3’ (reverse). The qualified PCR products were sequenced by Shanghai Sangon Biotech (Shanghai, China).

**Generation of knock-in rat**

The *Gnptab*-R587P knock-in rat model (Sprague Dawley) was generated by CRISPR/Cas9-mediated genome engineering in Cyagen Biosciences Inc. (Suzhou, China). Exon 13 of *Gnptab* gene (GenBank accession number: NM_001004164.2), in which the R587 is located, was selected as target site. The c.1760 G>C (CGT>CCT) will be introduced into exon 13 by donor oligo, in which targeting sequence is flanked by 120bp homologous sequences combined on both sides, through homology-directed recombination. Cas9 mRNA, sgRNA and donor oligo were co-injected into zygotes for knock-in rat production. The pups were genotyped by PCR combined with DNA sequencing. A female founder (F0) were generated, and then crossed to wild-type SD rat to breed F1 which were genotyped by PCR and DNA sequencing analysis. The sequences used for CRISPR/Cas9-mediated genome editing and the primers used for genotyping are listed in Supplementary Table S4. Rats were maintained in an SPF animal facility under the standard conditions.

**Supplemental References**

1. Li X, Shi W, Ding X, et al. Identification of a novel TBX5 mutation in a Chinese family with rare symptoms of Holt-Oram syndrome. *Heliyon*. 2022; 8(11):e11774.

**Supplemental Figures**


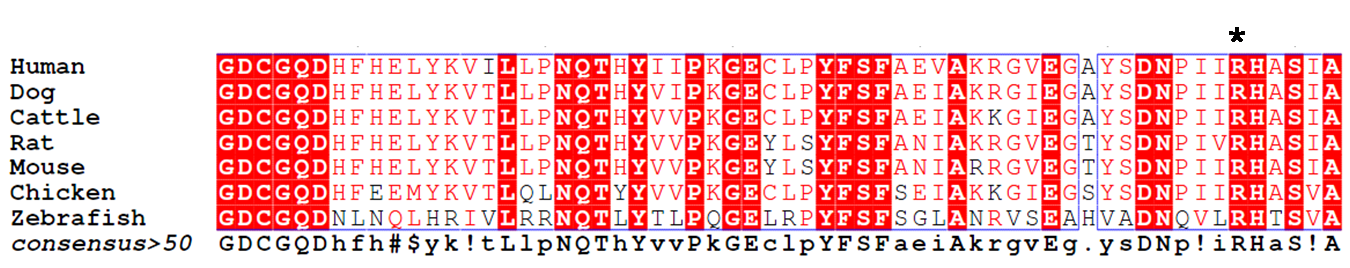


**Figure S1.** Alignment indicates that the amino acid (marked by star) affected by the mutation (c.1760G>C; p.R587P) in GNPTAB is evolutionarily conserved across species (<http://multalin.toulouse.inra.fr/multalin/>).


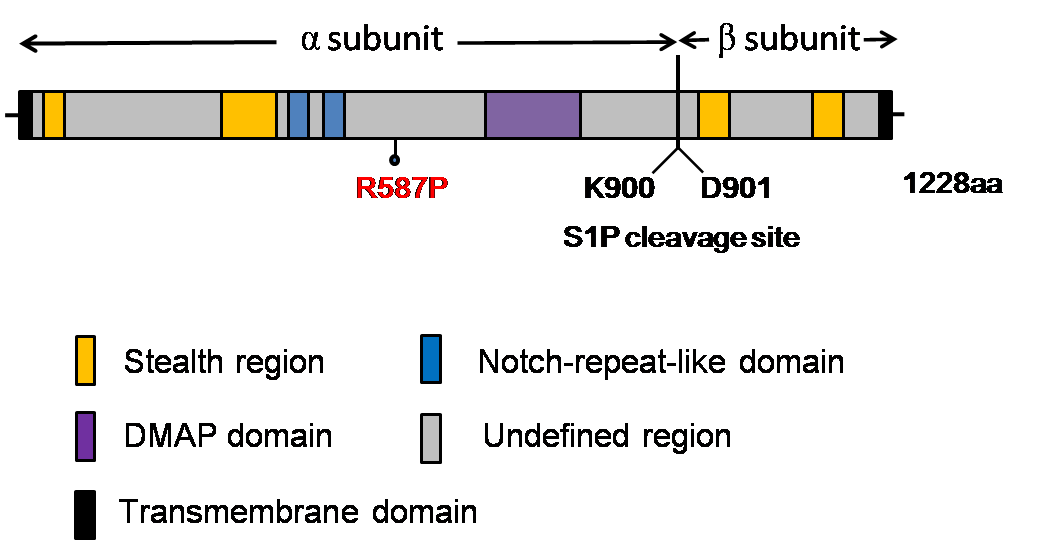


(B)

**Figure S2.** The schematic illustration of GNPTAB protein. The amino acid residue arginine 587 affected by mutation is located in the α-subunit of GlcNAc-phosphotransferase.


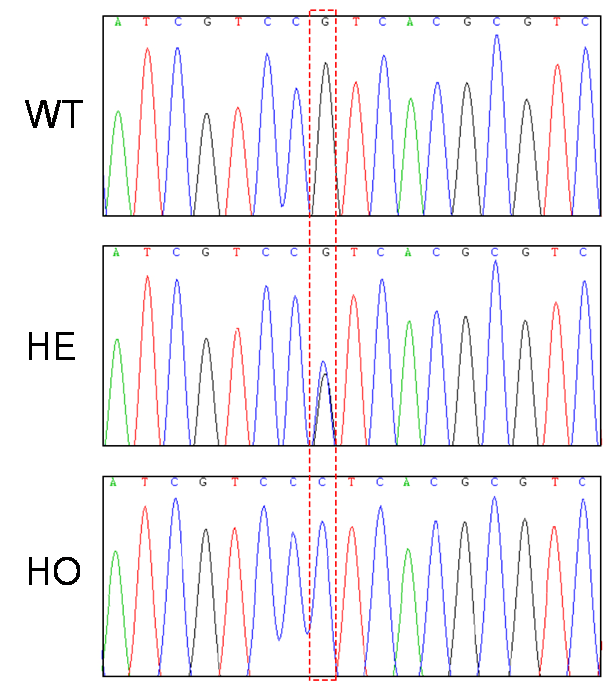


**Figure S3.** Genotyping of mutant rat embryos through Sanger sequencing. The mutation site is highlighted by red dashed line. WT, wild-type; HE, heterozygote (*Gnptab^R587P/+^*); HO, homozygote (*Gnptab^R587P/R587P^*).

**Supplemental Tables**

**Table S1.** The levels of MPS-related lysosomal enzyme activity in the patient’s plasma.

| **Lysosomal enzymes in plasma** | **Actual value** | **Normal value** |
| --- | --- | --- |
| A-L-idosinase (MPSI) | 33.7nmol/mg.h | 25.4-118.5nmol/mg.h |
| Idosaccharide-2-sulfatase (MPSII) | 81.6nmol/mg.4h | 30-120nmol/mg.4h |
| Galactosamine-6-sulfatase (MPSIVA) | 110.8nmol/mg.17h | 40-170nmol/mg.17h |
| B-D-galactosidase (MPSIVB) | 115.9nmol/mg.h | 50.3-140.7nmol/mg.h |
| Aryl sulfatase B (MPSVI) | 115.9nmol/mg.h | 50.4-175.2nmol/mg.h |
| β-glucuronidase (MPSVII) | 74.3nmol/mg.h | 38.1-202.5nmol/mg.h |

**Table S2.** The content of mucopolysaccharides in the patient’s urine.

| **Mucopolysaccharides in urine** | **Actual value** | **Normal value** |
| --- | --- | --- |
| Urine mucopolysaccharide creatinine ratio | 21.4mg/mmol | <29.4mg/mmol |
| Urine mucopolysaccharide electrophoresis (GAG) | （-） | （-） |
| Urine keratin sulfate (KS) | （-） | （-） |
| Urodermatin sulfate (DS) | （-） | （-） |
| Chondroitin sulfate in urine (CS) | （-） | （-） |

**Table S3.** Deleterious prediction of *GNPTAB* c.1760G>C mutation.

| **Gene** | **Transcript** | **Nucleotide change** | **Amino acid change** | **SIFT** | **FATHMM** | **PolyPhen2_HVAR** | **Mutation Taster** |
| --- | --- | --- | --- | --- | --- | --- | --- |
| *GNPTAB* | NM_024312 | c.G1760C | p.R587P | D | D | D | D |

Note: Chr, chromosome; SIFT, Sorting Intolerant From Tolerant; FATHMM, Functional Analysis Through Hidden Markov Models; PolyPhen2_HVAR, Polymorphism Phenotyping v2_HumanVar; D, deleterious.

**Table S4.** Sequences used for CRISPR/Cas9 editing and primers used for genotyping of *Gnptab-*R587P knock-in rats.

| **gRNA1 (matches reverse strand)** | GATAGACGCGTGACGGACGATGG |
| --- | --- |
| **gRNA2 (matches reverse strand)** | TAGACGCGTGACGGACGATGGGG |
| **Donor oligo** | AGCTTTGCAAACATAGCCAAAAGAGGCGTTGAGGGGACCTACAGTGACAACCCCATCGTCCCTCACGCGTCTATCGCAAACAAGTGGAAAACCATACACCTCATAATGCACAGTGGGATGAAC |
| **PCR genotyping-F** | GCCCGATGGTTGTCACTCTTTAATT |
| **PCR genotyping-R** | GGGAAGCGTTTCTCTTTGGGAA |
| **DNA sequencing primer** | TGAGGAAGAGGTGTCACTGGGC |
